# Hexa­kis­(3-chloro-2-methyl­anilinium) cyclo­hexa­phosphate dihydrate

**DOI:** 10.1107/S1600536813033230

**Published:** 2013-12-14

**Authors:** Raoudha Bel Haj Salah, Lamia Khederi, Mohamed Rzaigui

**Affiliations:** a Chemistry Laboratory of Materials, Sciences Faculty of Bizerta, 7021 Jarzouna, Bizerta, Tunisia

## Abstract

In the organic/inorganic salt hydrate, 6C_7_H_9_ClN^+^·P_6_O_18_^6−^·2H_2_O, the cyclo­hexa­phosphate anion resides on an inversion centre. The asymmetric unit consists of three cations, one half-anion and a water mol­ecule. In the crystal, the water mol­ecules and the [P_6_O_18_]^6−^ anions are linked by O—H⋯O hydrogen bonds, generating infinite layers parallel to the *ab* plane. These layers are inter­connected by the organic cations through N—H⋯O hydrogen bonds.

## Related literature   

For the properties of hybrid materials, see: Shi *et al.* (2000 ▶); Yokotani *et al.* (1989 ▶); Xiao *et al.* (2005 ▶); Koo *et al.* (2004 ▶). For related structures containing cyclo­hexa­phosphate rings, see: Khedhiri *et al.* (2012 ▶); Amri *et al.* (2009 ▶); Marouani & Rzaigui (2010 ▶); Averbuch-Pouchot & Durif (1991 ▶). For bond lengths, see: Fábry *et al.* (2002 ▶). For the preparation of cyclo­hexa­phospho­ric acid, see: Schülke & Kayser (1985 ▶).
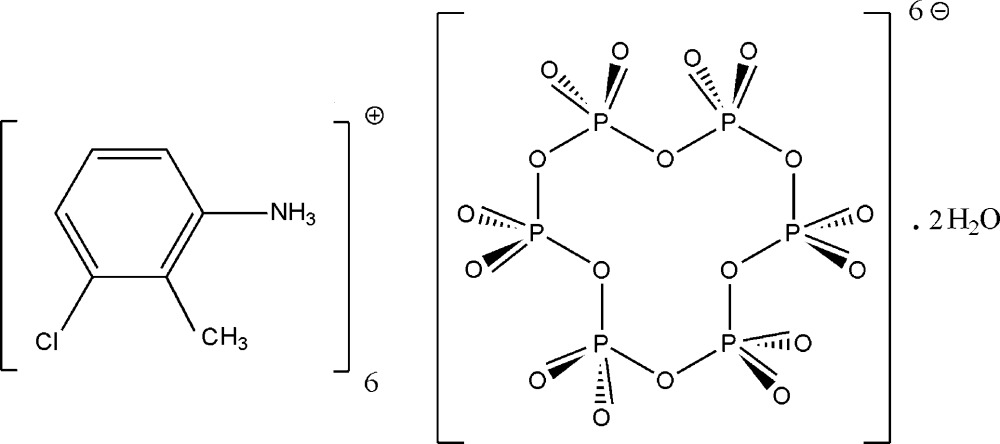


## Experimental   

### 

#### Crystal data  

6C_7_H_9_ClN^+^·P_6_O_18_^6−^·2H_2_O
*M* *_r_* = 1365.46
Triclinic, 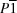

*a* = 9.576 (5) Å
*b* = 10.187 (4) Å
*c* = 17.392 (5) Å
α = 94.48 (2)°
β = 103.74 (4)°
γ = 112.25 (4)°
*V* = 1498.5 (12) Å^3^
*Z* = 1
Mo *K*α radiation
μ = 0.52 mm^−1^
*T* = 293 K
0.32 × 0.22 × 0.15 mm

#### Data collection  

Enraf–Nonius CAD-4 diffractometer
7464 measured reflections
7229 independent reflections
4800 reflections with *I* > 2σ(*I*)
*R*_int_ = 0.033
2 standard reflections every 120 min intensity decay: 5%

#### Refinement  

*R*[*F* ^2^ > 2σ(*F* ^2^)] = 0.050
*wR*(*F* ^2^) = 0.128
*S* = 1.02
7229 reflections
405 parameters
H atoms treated by a mixture of independent and constrained refinement
Δρ_max_ = 0.39 e Å^−3^
Δρ_min_ = −0.39 e Å^−3^

### 

Data collection: *CAD-4 EXPRESS* (Enraf–Nonius, 1994 ▶); cell refinement: *CAD-4 EXPRESS*; data reduction: *XCAD4* (Harms & Wocadlo, 1995 ▶); program(s) used to solve structure: *SHELXS97* (Sheldrick, 2008 ▶); program(s) used to refine structure: *SHELXL97* (Sheldrick, 2008 ▶); molecular graphics: *ORTEP-3 for Windows* (Farrugia, 2012 ▶); software used to prepare material for publication: *WinGX* (Farrugia, 2012 ▶).

## Supplementary Material

Crystal structure: contains datablock(s) I. DOI: 10.1107/S1600536813033230/fj2652sup1.cif

Structure factors: contains datablock(s) I. DOI: 10.1107/S1600536813033230/fj2652Isup2.hkl

Additional supporting information:  crystallographic information; 3D view; checkCIF report

## Figures and Tables

**Table 1:** Hydrogen-bond geometry (Å, °)

*D*—H⋯*A*	*D*—H	H⋯*A*	*D*⋯*A*	*D*—H⋯*A*
N1—H1*A*⋯O8	0.95 (4)	1.76 (4)	2.705 (4)	179 (5)
N1—H1*B*⋯O1*W* ^i^	0.97 (4)	1.85 (4)	2.779 (4)	160 (3)
N1—H1*C*⋯O4^ii^	0.94 (4)	1.83 (4)	2.766 (4)	173 (3)
O1*W*—H1*W*⋯O1^iii^	0.82 (5)	2.02 (6)	2.813 (4)	164 (5)
O1*W*—H2*W*⋯O5^ii^	0.82 (5)	2.14 (5)	2.934 (4)	163 (5)
N2—H2*A*⋯O5	0.90 (4)	2.02 (4)	2.871 (4)	156 (4)
N2—H2*B*⋯O2^iv^	0.93 (5)	1.85 (5)	2.763 (4)	166 (3)
N2—H2*C*⋯O1^v^	0.89 (4)	1.89 (4)	2.775 (4)	176 (4)
N3—H3*A*⋯O7^iv^	1.00 (5)	1.77 (5)	2.759 (4)	171 (5)
N3—H3*B*⋯O4^ii^	1.01 (4)	1.83 (4)	2.834 (4)	172 (4)
N3—H3*C*⋯O2^iv^	0.85 (4)	2.00 (4)	2.827 (4)	166 (3)

Symmetry codes: (i) 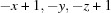; (ii) 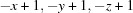; (iii) 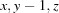; (iv) 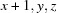; (v) 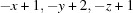.

**Fig. 1.**
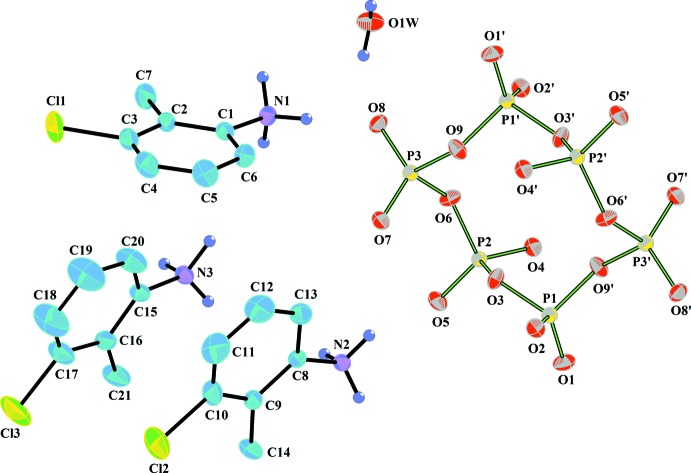
ORTEP drawing of (I) with displacement ellipsoids are drawn at the 30% probability level. [Symmetry code: (i) x, y, z]

**Fig. 2.**
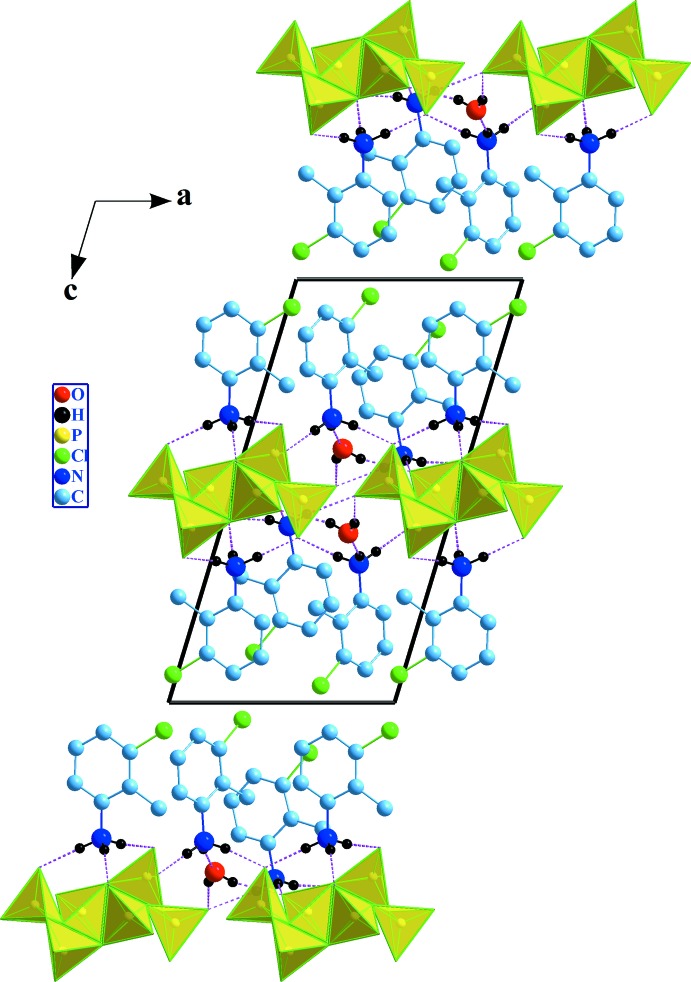
Structure projection of (I) along the b axis. The H-phenyl and H-methyl atoms are omitted for figure clarity. Hydrogen bonds are shown as dashed lines.

## supplementary crystallographic information

### 1. Comment

The interest of hybrid organic-inorganic materials has been continuously growing because of their potential applications in several fields (catalysis, biomoleculaire sciences and nonlinear optics) (Shi *et al.*, 2000, Yokotani *et al.*,1989). Interest of these compounds stems from the intriguing possibility of combining the features of both inorganic and organic systems within a single material (Koo *et al.*, 2004, Xiao *et al.*, 2005).

This paper reports synthesis and structural characterization of a new organic cyclohexaphosphate (I). The X-ray diffraction study of the title compound leads to the determination of its chemical formula. Configurations of the different organic and inorganic entities are depicted in the Figure 1. The examination of the atomic arrangement shows that the structure consists of inorganic layers made up from P_6_O_18_ rings and water molecules connected *via* hydrogen bonds and extended in the *ab* plane. The organic 3-chloro-2-methylammonium cations are displayed in the interlayer spaces compensating their negative charges and establishing H-bonds with the oxygen atoms of the anionic framework as shown in Figure 2.

The geometry of the phosphoric ring is commonly observed in other cyclohexaphosphates with a ring of low symmetry (Khedhiri *et al.*, 2012, Marouani *et al.*, 2010, Amri *et al.*, 2009). P—O distances range from 1.468 (2) to 1.606 (2) Å and O(*L*)—P—O(*L*) angles from 99.71 (11) to 102.22 (12)°. However, the P—P—P angles values of 97.92 (4), 104.2 (4) and 113.6 (4)° show that the P_6_O_18_ ring is significantly distorted from the ideal value 120°. Nevertheless, this distortion is comparatively less important than that observed in Cs_6_P_6_O_18_·6H_2_O, which shows the greatest distortion for the same angles, ranging between 93.2 and 145.5° (Averbuch-Pouchot & Durif, 1991).

The three crystallography distinct cations involved in this structure exhibit C—C and N—C and C—Cl distances in the range usually found in other molecule analogues such as 4-chloro-2-methylaniline (Fábry *et al.*, 2002). The C—C—C and C—C—N angles are similar to those expected for sp2 hybridization. These groups are almost planar with an average deviation of 0.0018. The mean geometric features of the hydrogen bonds show multiple kinds of hydrogen bonds. The first one involves O—H···O contacts, with O···O distances ranging from 2.813 (4) to 2.934 (4) Å, link between the phosphoric rings which form a bidimensional anionic framework, parallel to the *ab* plane (Fig. 2). While the second one includes N—H···O contacts, involving weak links since the N···O distances range from 2.705 (4) to 3.079 (4) Å, assuring the cohesion of the network. In addition, some H phenyl atoms also form weak C—H···O(N) interactions with the C···O(N)separations of 2.872 (5)–3.316 (5) Å. All these hydrogen bonds, Van Der Waals, and electrostatic interactions between organic cations and cyclohexaphosphate anions increase the structure stability in the title compound.

### 2. Experimental

Crystals of the title compound were synthesized by neutralization of an acidic aqueous solution of H_6_P_6_O_18_ (10 ml, 3.5 mmol) by adding dropwise a solution of 3-chloro-2-methylaniline (21 mmol in 20 ml of ethanol). The resulting solution is then kept at room temperature for several days to give colourless crystals of the title compound which are stable under normal conditions of temperature and humidity. The cyclohexaphosphoric acid used in this reaction was produced from an aqueous solution of Li_6_P_6_O_18_ (Schulke *et al.*, 1985) passed through an ion exchange resin (Amberlite IR120)

### 3. Refinement

Hydrogen atoms of the aromatic and methyl groups were placed at calculated positions with C—H = 0.93 and 0.96 Å, respectively and allowed to ride with *U*_iso_(H) = 1.5 *U*_eq_(C). H atoms on water molecules and the coordinates of the hydrogen atoms at the NH_3_ groups were located in Fourier difference maps and were refined freely simultaneously with individual *U*_iso_ values.

### Crystal data

**Table:**

6C_7_H_9_ClN^+^·P_6_O_18_^6^^−^·2H_2_O	*Z* = 1
*M**_r_* = 1365.46	*F*(000) = 704
Triclinic, *P*1	*D*_x_ = 1.513 Mg m^−^^3^
Hall symbol: -P 1	Mo *K*α radiation, λ = 0.71073 Å
*a* = 9.576 (5) Å	Cell parameters from 25 reflections
*b* = 10.187 (4) Å	θ = 9–11°
*c* = 17.392 (5) Å	µ = 0.52 mm^−^^1^
α = 94.48 (2)°	*T* = 293 K
β = 103.74 (4)°	Prism, colorless
γ = 112.25 (4)°	0.32 × 0.22 × 0.15 mm
*V* = 1498.5 (12) Å^3^	

### Data collection

**Table:**

Enraf–Nonius CAD-4 diffractometer	*R*_int_ = 0.033
Radiation source: fine-focus sealed tube	θ_max_ = 28.0°, θ_min_ = 2.2°
Graphite monochromator	*h* = −12→12
non–profiled ω scans	*k* = −13→13
7464 measured reflections	*l* = 0→22
7229 independent reflections	2 standard reflections every 120 min
4800 reflections with *I* > 2σ(*I*)	intensity decay: 5%

### Refinement

**Table:**

Refinement on *F*^2^	Primary atom site location: structure-invariant direct methods
Least-squares matrix: full	Secondary atom site location: difference Fourier map
*R*[*F*^2^ > 2σ(*F*^2^)] = 0.050	Hydrogen site location: inferred from neighbouring sites
*wR*(*F*^2^) = 0.128	H atoms treated by a mixture of independent and constrained refinement
*S* = 1.02	*w* = 1/[σ^2^(*F*_o_^2^) + (0.0596*P*)^2^ + 0.4583*P*] where *P* = (*F*_o_^2^ + 2*F*_c_^2^)/3
7229 reflections	(Δ/σ)_max_ = 0.001
405 parameters	Δρ_max_ = 0.39 e Å^−^^3^
0 restraints	Δρ_min_ = −0.39 e Å^−^^3^

### Special details

**Table:**

Geometry. All e.s.d.'s (except the e.s.d. in the dihedral angle between two l.s. planes) are estimated using the full covariance matrix. The cell e.s.d.'s are taken into account individually in the estimation of e.s.d.'s in distances, angles and torsion angles; correlations between e.s.d.'s in cell parameters are only used when they are defined by crystal symmetry. An approximate (isotropic) treatment of cell e.s.d.'s is used for estimating e.s.d.'s involving l.s. planes.
Refinement. Refinement of *F*^2^ against ALL reflections. The weighted *R*-factor *wR* and goodness of fit *S* are based on *F*^2^, conventional *R*-factors *R* are based on *F*, with *F* set to zero for negative *F*^2^. The threshold expression of *F*^2^ > σ(*F*^2^) is used only for calculating *R*-factors(gt) *etc*. and is not relevant to the choice of reflections for refinement. *R*-factors based on *F*^2^ are statistically about twice as large as those based on *F*, and *R*- factors based on ALL data will be even larger.

### Fractional atomic coordinates and isotropic or equivalent isotropic displacement parameters (Å^2^)

**Table:**

	*x*	*y*	*z*	*U*_iso_*/*U*_eq_	
P1	0.10693 (8)	0.76984 (7)	0.50600 (4)	0.0233 (2)	
P2	0.32227 (7)	0.62757 (7)	0.52138 (4)	0.0217 (2)	
P3	0.13627 (8)	0.34633 (7)	0.41193 (4)	0.0236 (2)	
O1	0.2396 (2)	0.9092 (2)	0.54611 (13)	0.0389 (7)	
O2	−0.0196 (2)	0.7621 (2)	0.43465 (12)	0.0320 (6)	
O3	0.1712 (2)	0.6560 (2)	0.47796 (11)	0.0302 (6)	
O4	0.3518 (2)	0.6539 (2)	0.61045 (11)	0.0301 (6)	
O5	0.4520 (2)	0.7054 (2)	0.48872 (13)	0.0363 (7)	
O6	0.2550 (2)	0.4574 (2)	0.49312 (12)	0.0333 (6)	
O7	0.1293 (2)	0.4211 (2)	0.34254 (12)	0.0329 (6)	
O8	0.1743 (2)	0.2189 (2)	0.41061 (12)	0.0343 (6)	
O9	0.0321 (2)	0.6948 (2)	0.57240 (12)	0.0327 (6)	
Cl1	0.34937 (16)	−0.02530 (14)	0.04005 (7)	0.0821 (5)	
N1	0.3436 (3)	0.1365 (3)	0.32844 (15)	0.0306 (8)	
C1	0.2839 (3)	0.1210 (3)	0.24115 (17)	0.0308 (8)	
C2	0.3467 (4)	0.0618 (3)	0.19071 (19)	0.0375 (10)	
C3	0.2856 (4)	0.0540 (4)	0.1089 (2)	0.0483 (11)	
C4	0.1740 (5)	0.1046 (4)	0.0786 (2)	0.0564 (11)	
C5	0.1155 (4)	0.1631 (4)	0.1303 (2)	0.0560 (14)	
C6	0.1694 (4)	0.1698 (3)	0.21233 (19)	0.0416 (10)	
C7	0.4738 (5)	0.0117 (4)	0.2232 (2)	0.0579 (16)	
Cl2	0.6890 (2)	0.81050 (16)	0.11837 (7)	0.1000 (6)	
N2	0.7057 (3)	0.8023 (3)	0.41707 (15)	0.0281 (8)	
C8	0.6282 (3)	0.7400 (3)	0.33153 (17)	0.0292 (8)	
C9	0.6979 (4)	0.8032 (3)	0.27466 (18)	0.0359 (10)	
C10	0.6160 (5)	0.7370 (4)	0.1948 (2)	0.0546 (13)	
C11	0.4744 (6)	0.6175 (5)	0.1737 (3)	0.0785 (18)	
C12	0.4108 (5)	0.5590 (5)	0.2319 (3)	0.0726 (17)	
C13	0.4874 (4)	0.6200 (4)	0.3115 (2)	0.0464 (11)	
C14	0.8500 (4)	0.9359 (4)	0.2981 (2)	0.0518 (12)	
Cl3	1.0171 (2)	0.67872 (17)	0.06613 (8)	0.1091 (7)	
N3	0.8848 (3)	0.5080 (3)	0.32105 (15)	0.0317 (8)	
C15	0.8371 (3)	0.4984 (3)	0.23430 (18)	0.0345 (9)	
C16	0.9415 (4)	0.5911 (3)	0.19903 (19)	0.0403 (10)	
C17	0.8953 (5)	0.5702 (4)	0.1164 (2)	0.0641 (14)	
C18	0.7528 (7)	0.4644 (6)	0.0704 (3)	0.109 (2)	
C19	0.6514 (6)	0.3770 (6)	0.1070 (3)	0.105 (2)	
C20	0.6930 (4)	0.3946 (4)	0.1898 (2)	0.0662 (12)	
C21	1.0981 (4)	0.7057 (4)	0.2497 (3)	0.0616 (14)	
O1W	0.5687 (3)	0.0657 (3)	0.59977 (17)	0.0498 (9)	
H1A	0.284 (4)	0.166 (4)	0.357 (2)	0.056 (11)*	
H1B	0.350 (4)	0.050 (4)	0.346 (2)	0.049 (10)*	
H1C	0.446 (4)	0.210 (4)	0.3448 (19)	0.039 (9)*	
H4	0.13811	0.09937	0.02333	0.0851*	
H5	0.03996	0.19805	0.11032	0.0839*	
H6	0.12852	0.20698	0.24769	0.0624*	
H7A	0.49965	0.02657	0.28092	0.0874*	
H7B	0.43730	−0.08912	0.20193	0.0874*	
H7C	0.56571	0.06563	0.20757	0.0874*	
H2A	0.640 (4)	0.755 (4)	0.445 (2)	0.047 (10)*	
H2B	0.797 (5)	0.786 (4)	0.431 (2)	0.059 (11)*	
H2C	0.726 (4)	0.896 (4)	0.427 (2)	0.043 (9)*	
H11	0.42203	0.57662	0.11971	0.1168*	
H12	0.31566	0.47788	0.21759	0.1088*	
H13	0.44471	0.58085	0.35145	0.0694*	
H14A	0.88732	0.96426	0.35569	0.0779*	
H14B	0.92709	0.91552	0.27887	0.0779*	
H14C	0.83286	1.01277	0.27464	0.0779*	
H3A	0.979 (5)	0.484 (5)	0.334 (3)	0.089 (15)*	
H3B	0.802 (5)	0.442 (4)	0.344 (2)	0.066 (12)*	
H3C	0.912 (4)	0.591 (4)	0.348 (2)	0.058 (12)*	
H18	0.72554	0.45254	0.01451	0.1635*	
H19	0.55476	0.30596	0.07609	0.1573*	
H20	0.62411	0.33662	0.21526	0.0991*	
H21A	1.10761	0.70244	0.30568	0.0919*	
H21B	1.18180	0.68856	0.23587	0.0919*	
H21C	1.10459	0.79895	0.23990	0.0919*	
H1W	0.476 (6)	0.015 (5)	0.576 (3)	0.085 (17)*	
H2W	0.583 (5)	0.135 (5)	0.577 (3)	0.083 (16)*	

### Atomic displacement parameters (Å^2^)

**Table:**

	*U*^11^	*U*^22^	*U*^33^	*U*^12^	*U*^13^	*U*^23^
P1	0.0201 (3)	0.0222 (3)	0.0295 (4)	0.0093 (3)	0.0095 (3)	0.0054 (3)
P2	0.0176 (3)	0.0215 (3)	0.0254 (4)	0.0070 (3)	0.0073 (3)	0.0033 (3)
P3	0.0206 (3)	0.0227 (3)	0.0275 (4)	0.0079 (3)	0.0090 (3)	0.0033 (3)
O1	0.0334 (11)	0.0220 (10)	0.0539 (14)	0.0051 (9)	0.0112 (10)	0.0025 (9)
O2	0.0303 (10)	0.0385 (11)	0.0341 (11)	0.0197 (9)	0.0102 (9)	0.0125 (9)
O3	0.0270 (10)	0.0348 (11)	0.0308 (10)	0.0192 (9)	0.0038 (8)	−0.0019 (8)
O4	0.0260 (10)	0.0332 (10)	0.0258 (10)	0.0085 (8)	0.0053 (8)	0.0021 (8)
O5	0.0253 (10)	0.0401 (12)	0.0422 (12)	0.0070 (9)	0.0173 (9)	0.0110 (10)
O6	0.0344 (11)	0.0223 (10)	0.0355 (11)	0.0100 (9)	0.0005 (9)	0.0018 (8)
O7	0.0339 (11)	0.0351 (11)	0.0329 (11)	0.0134 (9)	0.0153 (9)	0.0102 (9)
O8	0.0363 (11)	0.0281 (10)	0.0425 (12)	0.0163 (9)	0.0142 (9)	0.0032 (9)
O9	0.0232 (9)	0.0468 (12)	0.0355 (11)	0.0167 (9)	0.0146 (8)	0.0169 (9)
Cl1	0.1113 (10)	0.0872 (8)	0.0560 (6)	0.0345 (7)	0.0527 (7)	0.0022 (6)
N1	0.0265 (13)	0.0326 (14)	0.0310 (13)	0.0116 (11)	0.0078 (10)	0.0014 (11)
C1	0.0283 (14)	0.0289 (14)	0.0277 (14)	0.0048 (12)	0.0073 (12)	0.0018 (12)
C2	0.0372 (16)	0.0324 (16)	0.0406 (18)	0.0076 (13)	0.0194 (14)	0.0029 (13)
C3	0.055 (2)	0.0407 (19)	0.0402 (19)	0.0060 (16)	0.0233 (17)	−0.0002 (15)
C4	0.057 (2)	0.066 (2)	0.0317 (18)	0.013 (2)	0.0083 (17)	0.0068 (17)
C5	0.046 (2)	0.070 (3)	0.044 (2)	0.0226 (19)	0.0013 (17)	0.0090 (18)
C6	0.0369 (17)	0.0464 (19)	0.0375 (18)	0.0168 (15)	0.0061 (14)	0.0010 (14)
C7	0.065 (2)	0.068 (3)	0.068 (3)	0.042 (2)	0.040 (2)	0.023 (2)
Cl2	0.1609 (14)	0.1171 (11)	0.0480 (6)	0.0661 (10)	0.0541 (8)	0.0379 (7)
N2	0.0295 (13)	0.0284 (13)	0.0300 (13)	0.0137 (11)	0.0109 (11)	0.0083 (10)
C8	0.0295 (14)	0.0285 (14)	0.0311 (15)	0.0142 (12)	0.0078 (12)	0.0051 (12)
C9	0.0436 (17)	0.0363 (16)	0.0364 (17)	0.0218 (14)	0.0167 (14)	0.0103 (13)
C10	0.078 (3)	0.063 (2)	0.0319 (18)	0.035 (2)	0.0210 (18)	0.0123 (17)
C11	0.094 (4)	0.076 (3)	0.039 (2)	0.025 (3)	−0.005 (2)	−0.010 (2)
C12	0.062 (3)	0.058 (3)	0.057 (3)	−0.002 (2)	−0.004 (2)	−0.011 (2)
C13	0.0406 (18)	0.0370 (18)	0.050 (2)	0.0044 (15)	0.0136 (16)	0.0016 (15)
C14	0.051 (2)	0.047 (2)	0.062 (2)	0.0133 (17)	0.0319 (18)	0.0225 (18)
Cl3	0.1496 (14)	0.1094 (11)	0.0706 (8)	0.0299 (10)	0.0648 (9)	0.0485 (8)
N3	0.0322 (14)	0.0333 (14)	0.0278 (13)	0.0114 (12)	0.0081 (11)	0.0076 (11)
C15	0.0356 (16)	0.0332 (16)	0.0313 (15)	0.0114 (13)	0.0074 (13)	0.0090 (12)
C16	0.0448 (18)	0.0356 (17)	0.0379 (17)	0.0138 (14)	0.0111 (15)	0.0092 (14)
C17	0.080 (3)	0.059 (2)	0.040 (2)	0.011 (2)	0.020 (2)	0.0190 (18)
C18	0.126 (5)	0.109 (4)	0.033 (2)	−0.002 (4)	0.002 (3)	0.012 (3)
C19	0.086 (4)	0.101 (4)	0.050 (3)	−0.022 (3)	−0.015 (3)	0.001 (3)
C20	0.050 (2)	0.061 (2)	0.049 (2)	−0.0095 (19)	−0.0008 (18)	0.0114 (19)
C21	0.050 (2)	0.051 (2)	0.068 (3)	−0.0003 (18)	0.022 (2)	0.017 (2)
O1W	0.0428 (15)	0.0427 (14)	0.0590 (16)	0.0150 (12)	0.0074 (13)	0.0182 (13)

### Geometric parameters (Å, º)

**Table:**

Cl1—C3	1.741 (4)	C4—C5	1.373 (6)
Cl2—C10	1.737 (4)	C5—C6	1.384 (5)
Cl3—C17	1.734 (5)	C4—H4	0.9300
P1—O2	1.487 (2)	C5—H5	0.9300
P1—O9	1.596 (2)	C6—H6	0.9300
P1—O3	1.599 (2)	C7—H7C	0.9600
P1—O1	1.475 (2)	C7—H7B	0.9600
P2—O5	1.468 (2)	C7—H7A	0.9600
P2—O3	1.606 (2)	C8—C9	1.388 (4)
P2—O4	1.490 (2)	C8—C13	1.377 (5)
P2—O6	1.591 (2)	C9—C14	1.502 (5)
P3—O9^i^	1.601 (2)	C9—C10	1.391 (5)
P3—O7	1.478 (2)	C10—C11	1.378 (7)
P3—O8	1.475 (2)	C11—C12	1.365 (7)
P3—O6	1.606 (2)	C12—C13	1.371 (6)
O1W—H1W	0.82 (5)	C11—H11	0.9300
O1W—H2W	0.82 (5)	C12—H12	0.9300
N1—C1	1.462 (4)	C13—H13	0.9300
N1—H1C	0.94 (4)	C14—H14C	0.9600
N1—H1B	0.97 (4)	C14—H14A	0.9600
N1—H1A	0.95 (4)	C14—H14B	0.9600
N2—C8	1.462 (4)	C15—C16	1.391 (5)
N2—H2B	0.93 (5)	C15—C20	1.372 (5)
N2—H2A	0.90 (4)	C16—C21	1.508 (6)
N2—H2C	0.89 (4)	C16—C17	1.373 (5)
N3—C15	1.453 (4)	C17—C18	1.377 (7)
N3—H3C	0.85 (4)	C18—C19	1.367 (8)
N3—H3B	1.01 (4)	C19—C20	1.378 (6)
N3—H3A	1.00 (5)	C18—H18	0.9300
C1—C6	1.375 (5)	C19—H19	0.9300
C1—C2	1.392 (5)	C20—H20	0.9300
C2—C7	1.500 (6)	C21—H21C	0.9600
C2—C3	1.387 (5)	C21—H21A	0.9600
C3—C4	1.370 (6)	C21—H21B	0.9600
			
O1—P1—O2	120.42 (13)	C5—C6—H6	120.00
O1—P1—O3	110.23 (13)	C1—C6—H6	120.00
O1—P1—O9	108.66 (12)	C2—C7—H7C	109.00
O2—P1—O3	105.54 (12)	C2—C7—H7A	109.00
O2—P1—O9	110.27 (13)	C2—C7—H7B	109.00
O3—P1—O9	99.71 (11)	H7A—C7—H7B	109.00
O3—P2—O4	110.07 (12)	H7A—C7—H7C	109.00
O3—P2—O5	108.64 (12)	H7B—C7—H7C	109.00
O3—P2—O6	100.22 (12)	N2—C8—C13	117.5 (3)
O4—P2—O5	118.09 (13)	C9—C8—C13	123.1 (3)
O4—P2—O6	106.34 (12)	N2—C8—C9	119.4 (3)
O5—P2—O6	112.00 (12)	C10—C9—C14	122.5 (3)
O6—P3—O7	110.89 (12)	C8—C9—C14	122.0 (3)
O6—P3—O8	106.05 (12)	C8—C9—C10	115.5 (3)
O6—P3—O9^i^	102.62 (12)	Cl2—C10—C9	119.5 (3)
O7—P3—O8	121.53 (13)	C9—C10—C11	122.1 (4)
O7—P3—O9^i^	104.67 (12)	Cl2—C10—C11	118.4 (3)
O8—P3—O9^i^	109.53 (12)	C10—C11—C12	120.2 (4)
P1—O3—P2	130.72 (13)	C11—C12—C13	119.9 (5)
P2—O6—P3	134.20 (14)	C8—C13—C12	119.2 (4)
P1—O9—P3^i^	133.29 (14)	C12—C11—H11	120.00
H1W—O1W—H2W	101 (5)	C10—C11—H11	120.00
H1A—N1—H1B	108 (3)	C13—C12—H12	120.00
H1A—N1—H1C	107 (3)	C11—C12—H12	120.00
C1—N1—H1B	114 (2)	C8—C13—H13	120.00
C1—N1—H1C	107 (2)	C12—C13—H13	120.00
H1B—N1—H1C	108 (3)	C9—C14—H14C	109.00
C1—N1—H1A	113 (2)	C9—C14—H14B	109.00
H2B—N2—H2C	112 (4)	H14A—C14—H14C	109.00
H2A—N2—H2B	110 (3)	C9—C14—H14A	109.00
H2A—N2—H2C	109 (4)	H14A—C14—H14B	109.00
C8—N2—H2A	108 (2)	H14B—C14—H14C	109.00
C8—N2—H2B	107 (2)	C16—C15—C20	122.3 (3)
C8—N2—H2C	111 (2)	N3—C15—C16	118.9 (3)
C15—N3—H3B	116 (2)	N3—C15—C20	118.8 (3)
H3B—N3—H3C	104 (3)	C15—C16—C21	121.1 (3)
C15—N3—H3C	114 (2)	C17—C16—C21	122.5 (4)
C15—N3—H3A	107 (3)	C15—C16—C17	116.4 (3)
H3A—N3—H3C	107 (4)	Cl3—C17—C18	117.5 (3)
H3A—N3—H3B	109 (4)	C16—C17—C18	122.2 (4)
C2—C1—C6	122.5 (3)	Cl3—C17—C16	120.3 (3)
N1—C1—C6	117.9 (3)	C17—C18—C19	119.9 (5)
N1—C1—C2	119.6 (3)	C18—C19—C20	119.7 (5)
C3—C2—C7	122.4 (3)	C15—C20—C19	119.3 (4)
C1—C2—C7	121.9 (3)	C17—C18—H18	120.00
C1—C2—C3	115.7 (3)	C19—C18—H18	120.00
Cl1—C3—C2	119.6 (3)	C20—C19—H19	120.00
C2—C3—C4	123.0 (4)	C18—C19—H19	120.00
Cl1—C3—C4	117.4 (3)	C15—C20—H20	120.00
C3—C4—C5	119.6 (3)	C19—C20—H20	120.00
C4—C5—C6	119.7 (4)	C16—C21—H21B	109.00
C1—C6—C5	119.5 (3)	C16—C21—H21C	109.00
C5—C4—H4	120.00	C16—C21—H21A	109.00
C3—C4—H4	120.00	H21A—C21—H21C	109.00
C4—C5—H5	120.00	H21B—C21—H21C	110.00
C6—C5—H5	120.00	H21A—C21—H21B	109.00
			
O1—P1—O3—P2	38.7 (2)	C3—C4—C5—C6	−0.3 (6)
O2—P1—O3—P2	170.22 (16)	C4—C5—C6—C1	1.6 (5)
O9—P1—O3—P2	−75.42 (19)	N2—C8—C9—C10	−179.9 (3)
O2—P1—O9—P3^i^	−19.0 (2)	N2—C8—C9—C14	−1.1 (5)
O3—P1—O9—P3^i^	−129.62 (18)	C13—C8—C9—C10	−0.1 (5)
O1—P1—O9—P3^i^	115.04 (19)	C13—C8—C9—C14	178.7 (4)
O4—P2—O3—P1	33.9 (2)	N2—C8—C13—C12	179.7 (4)
O5—P2—O3—P1	−96.79 (18)	C9—C8—C13—C12	−0.2 (6)
O5—P2—O6—P3	−79.6 (2)	C8—C9—C10—Cl2	177.9 (3)
O6—P2—O3—P1	145.66 (17)	C8—C9—C10—C11	0.6 (6)
O3—P2—O6—P3	35.4 (2)	C14—C9—C10—Cl2	−1.0 (6)
O4—P2—O6—P3	150.01 (19)	C14—C9—C10—C11	−178.3 (4)
O7—P3—O6—P2	22.7 (2)	Cl2—C10—C11—C12	−178.1 (4)
O8—P3—O6—P2	156.50 (19)	C9—C10—C11—C12	−0.8 (8)
O9^i^—P3—O6—P2	−88.6 (2)	C10—C11—C12—C13	0.5 (8)
O6^i^—P3^i^—O9—P1	61.0 (2)	C11—C12—C13—C8	−0.1 (7)
O7^i^—P3^i^—O9—P1	176.90 (17)	N3—C15—C16—C17	175.3 (3)
O8^i^—P3^i^—O9—P1	−51.3 (2)	N3—C15—C16—C21	−3.4 (5)
N1—C1—C2—C3	−178.7 (3)	C20—C15—C16—C17	−2.3 (5)
N1—C1—C2—C7	0.6 (5)	C20—C15—C16—C21	179.0 (4)
C6—C1—C2—C3	−0.4 (5)	N3—C15—C20—C19	−175.0 (4)
C6—C1—C2—C7	178.8 (3)	C16—C15—C20—C19	2.5 (6)
N1—C1—C6—C5	177.0 (3)	C15—C16—C17—Cl3	−179.5 (3)
C2—C1—C6—C5	−1.3 (5)	C15—C16—C17—C18	0.6 (7)
C1—C2—C3—Cl1	−177.2 (3)	C21—C16—C17—Cl3	−0.8 (6)
C1—C2—C3—C4	1.9 (5)	C21—C16—C17—C18	179.3 (5)
C7—C2—C3—Cl1	3.6 (5)	Cl3—C17—C18—C19	−179.2 (5)
C7—C2—C3—C4	−177.3 (4)	C16—C17—C18—C19	0.7 (9)
Cl1—C3—C4—C5	177.5 (3)	C17—C18—C19—C20	−0.5 (9)
C2—C3—C4—C5	−1.6 (6)	C18—C19—C20—C15	−1.0 (8)

Symmetry code: (i) −*x*, −*y*+1, −*z*+1.

### Hydrogen-bond geometry (Å, º)

**Table:**

*D*—H···*A*	*D*—H	H···*A*	*D*···*A*	*D*—H···*A*
N1—H1*A*···O8	0.95 (4)	1.76 (4)	2.705 (4)	179 (5)
N1—H1*B*···O1*W*^ii^	0.97 (4)	1.85 (4)	2.779 (4)	160 (3)
N1—H1*C*···O4^iii^	0.94 (4)	1.83 (4)	2.766 (4)	173 (3)
O1*W*—H1*W*···O1^iv^	0.82 (5)	2.02 (6)	2.813 (4)	164 (5)
O1*W*—H2*W*···O5^iii^	0.82 (5)	2.14 (5)	2.934 (4)	163 (5)
N2—H2*A*···O5	0.90 (4)	2.02 (4)	2.871 (4)	156 (4)
N2—H2*B*···O2^v^	0.93 (5)	1.85 (5)	2.763 (4)	166 (3)
N2—H2*C*···O1^vi^	0.89 (4)	1.89 (4)	2.775 (4)	176 (4)
N3—H3*A*···O7^v^	1.00 (5)	1.77 (5)	2.759 (4)	171 (5)
N3—H3*B*···O4^iii^	1.01 (4)	1.83 (4)	2.834 (4)	172 (4)
N3—H3*C*···O2^v^	0.85 (4)	2.00 (4)	2.827 (4)	166 (3)

Symmetry codes: (ii) −*x*+1, −*y*, −*z*+1; (iii) −*x*+1, −*y*+1, −*z*+1; (iv) *x*, *y*−1, *z*; (v) *x*+1, *y*, *z*; (vi) −*x*+1, −*y*+2, −*z*+1.
